# Regulation of the cold-sensing TRPM8 channels by phosphoinositides and G_q_-coupled receptors

**DOI:** 10.1080/19336950.2020.1734266

**Published:** 2020-02-26

**Authors:** Luyu Liu, Tibor Rohacs

**Affiliations:** Department of Pharmacology, Physiology and Neuroscience, Rutgers New Jersey Medical School, Newark, NJ, USA

**Keywords:** TRPM8, PIP2, phosphoinositides, GPCR, G-alpha-q

## Abstract

The Transient Receptor Potential Melastatin 8 (TRPM8) ion channel is an important sensor of environmental cold temperatures. Cold- and menthol-induced activation of this channel requires the presence of the membrane phospholipid phosphatidylinositol 4,5-bisphosphate [PI(4,5)P_2_]. This review discusses recent findings on the role of PI(4,5)P_2_ and G-proteins in the modulation of TRPM8 upon receptor activation. We will also summarize knowledge on the role of PI(4,5)P_2_ in Ca^2+^ dependent desensitization/adaptation of TRPM8 activity, and recent advances in the structural basis of how this lipid binds to TRPM8.

## Introduction

Peppermint oil, and its main active compound, menthol has been known to evoke a cooling sensation for centuries [1]. The primary menthol receptor, the Transient Receptor Potential Melastatin 8 (TRPM8) channel was identified independently by two laboratories in 2002 [2,3]. This channel is expressed in the sensory neurons of the Dorsal Root Ganglia (DRG) and the Trigeminal Ganglia (TG), and it is activated by cold, and chemical agonists such as menthol, icilin and WS12 [4,5]. TRPM8 is a Ca^2+^ permeable nonselective cation channel; its activation depolarizes neurons and induces a Ca^2+^ signal. Genetic deletion of TRPM8 in mice revealed that this channel is important for sensing moderately cold temperatures [6–8]. Later studies showed that the channel is also involved in pathological cold sensation [9,10].

TRPM8 activity decreases in the continuous presence of menthol in an extracellular Ca^2+^ dependent manner [2]. This phenomenon was initially termed desensitization [2], and later referred to as adaptation [11]; here we will use these two terms interchangeably. This decreased activity was proposed to be caused by Ca^2+^ dependent activation of a phospholipase C (PLC) isoform by influx of Ca^2+^ through the channel, and the subsequent reduction in the levels of the plasma membrane phospholipid phosphatidylinositol 4,5-bisphosphate [PI(4,5)P_2_], which is an important cofactor for TRPM8 activity [12]. This mechanism is widely supported by work from several laboratories; here we will briefly discuss the evidence for the involvement of PI(4,5)P_2_ in channel desensitization, as well as the recent structural advances in the molecular mechanism of PI(4,5)P_2_ activation of TRPM8.

PLC activation by G-Protein Coupled Receptors (GPCRs) also inhibits TRPM8 activity. Many of the G_q_-coupled receptors in DRG neurons are activated by proinflammatory mediators such as bradykinin, prostaglandins, histamine and extracellular ATP. Activation of these receptors is known to sensitize the heat- and capsaicin-activated Transient Receptor Potential Vanilloid 1 (TRPV1) channels, leading to increased sensitivity to heat (thermal hyperalgesia) [13–15]. Inhibition of TRPM8 by G_q_-coupled receptor activation was proposed to contribute to thermal hyperalgesia in inflammation [16]. The focus of this review will be to discuss the involvement of PI(4,5)P_2_, and alternative mechanisms in GPCR-mediated inhibition of TRPM8, discussing in detail two recent publications that suggested seemingly contradictory conclusions [17,18].

## PI(4,5)P_2_ is a specific direct regulator of TRPM8 activity

**Figure 1.**
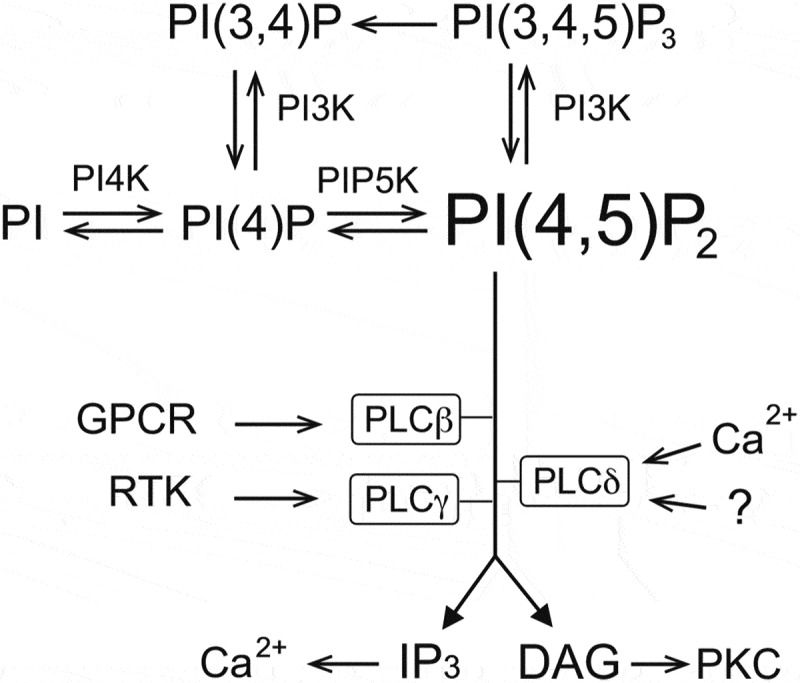
Phosphoinositides metabolism, see main text for details.

PI(4,5)P_2_ constitutes ~1% of the lipids in the inner leaflet of the plasma membrane. It is generated by the sequential phosphorylation of phosphatidylinositol (PI) to PI(4)P and then to PI(4,5)P_2_, catalyzed by phosphatidylinositol 4-kinase (PI4K) and phosphatidylinositol 4-phosphate 5-kinase (PIP5K) enzymes respectively (Figure 1). PI(4,5)P_2_ and PI(4)P can also be further phosphorylated by phosphoinositide 3-kinase (PI3K) enzymes to PI(3,4,5)P_3_ and PI(3,4)P_2_ respectively. PLC enzymes convert PI(4,5)P_2_ into diacylglycerol (DAG), which activates Protein Kinase C (PKC) and inositol 1,4,5 trisphosphate (IP_3_), which releases Ca^2+^ from the endoplasmic reticulum. PLCβ enzymes are activated by G-protein coupled receptors that couple to G_αq_, while PLCγ-s are activated by receptor tyrosine kinases [19]. All PLC isoforms require some Ca^2+^ for activity; PLCδ enzymes are the most Ca^2+^ sensitive isoforms, and they can be activated by Ca^2+^ alone in a cellular environment (Figure 1).

There are multiple lines of evidence indicating that PI(4,5)P_2_ is required for menthol- and cold-induced activity of TRPM8; most of these experiments have been discussed in detail before [20], we briefly review them here. First, two laboratories independently demonstrated that TRPM8 activity shows a spontaneous decrease (rundown) in excised inside out patches [12,21], which is a common property of PI(4,5)P_2_ dependent ion channels, and it is generally attributed to dephosphorylation of PI(4,5)P_2_ to PI(4)P and PI [22]. Rundown was accelerated by the application of poly-Lys, which chelates PI(4,5)P_2_ [12] and was delayed by a phosphatase inhibitor cocktail, that prevents dephosphorylation of PI(4,5)P_2_ [21]. More importantly, channel activity was restored in excised patches by the application of PI(4,5)P_2_ [12,21]; while other phosphoinositides, such as PI(4)P, PI(3,4)P_2_ and PI(3,4,5)P_3_ were much less effective, exerting 10–30% of the effect of PI(4,5)P_2_ [12]. TRPM8 activity was also restored by the application of MgATP in excised inside out patches, and its effect was eliminated by blocking PI4K activity in the patch [23], signifying that MgATP exerted its effect via replenishing PI(4,5)P_2_. The purified TRPM8 protein reconstituted in planar lipid bilayers also required PI(4,5)P_2_ for cold- or menthol-induced activity [24]. Similar to excised patches, PI(4)P, PI(3,4)P_2_ and PI(3,4,5)P_3_ were less effective than PI(4,5)P_2_ in planar lipid bilayers [24]. The ability of PI(4,5)P_2_ to support the activity of the purified TRPM8 protein in planar lipid bilayers demonstrates that it exerts its effect by binding to the channel directly.

**Figure 2.**
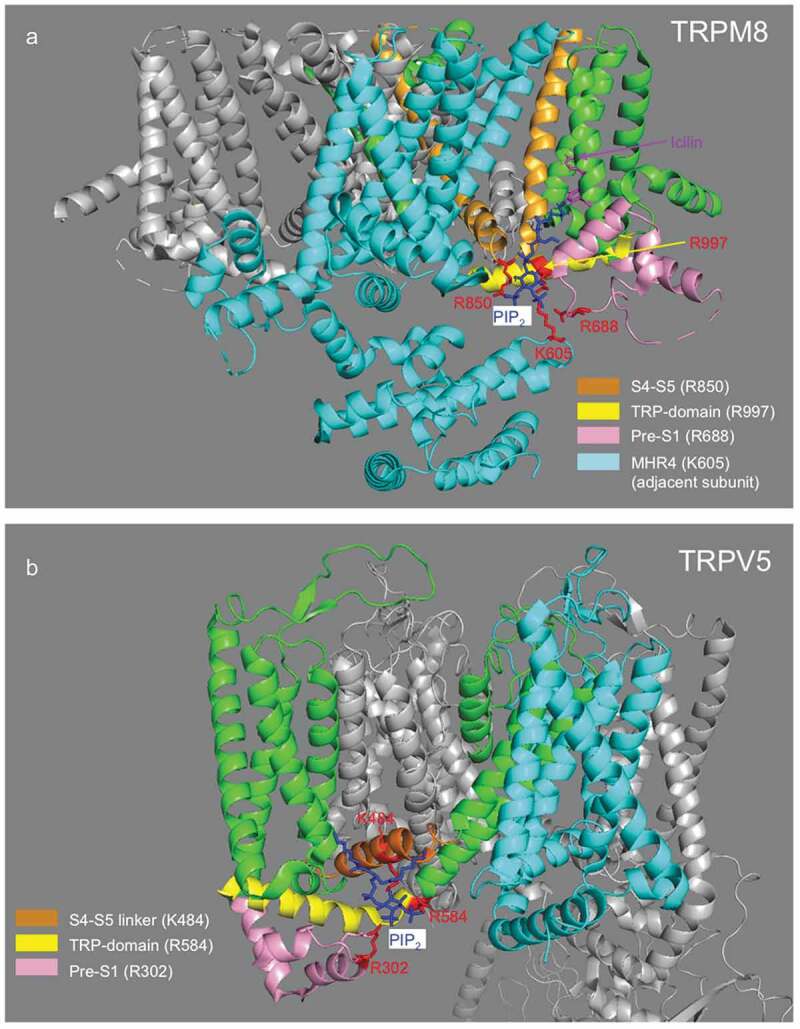
Structure of TRPM8 and TRPV5 with PI(4,5)P_2_. (a). Structure of TRPM8 with PI(4,5)P_2_ and icilin (6nr3) from Yin et al [25]. PI(4,5)P_2_ is colored blue, interacting residues are colored red. Parts of the channel where the interacting residues located are also color-coded: S4-S5 orange, TRP-domain yellow, Pres-S1 segment purple. The remaining parts (S6 and S1-S3) of the same subunit where these residues are located are colored green. The entire adjacent subunit is labeled cyan, the MHR4 region of this subunit contains an additional PI(4,5)P_2_ interacting residue; most other cytoplasmic parts have been removed for clarity. (b). Structure of TRPV5 with PI(4,5)P_2_ (6dmu) from Hughes et al [27]. Color-coding is similar to that in panel A.

The recent cryoEM structure of TRPM8 with PI(4,5)P_2_ and icilin (and WS12) gives a detailed molecular understanding of how the lipid binds to TRPM8 (Figure 2). Positively charged residues from three regions of the same subunit (TRP domain, S4-S5 linker and preS1 segment), and one additional cytoplasmic region (MHR4) of an adjacent subunit form the lipid binding site in the TRPM8-PI(4,5)P_2_ (6nr3) structure [25]. Consistent with being a PI(4,5)P_2_ contact residue, neutralization of the positively charged R998 residue in the proximal C-terminal TRP domain of the rat TRPM8 (R997 in flycatcher isoform used for structure determination) was shown earlier to reduce the apparent affinity of PI(4,5)P_2_ in excised inside out patch clamp experiments [12]. Neutralization of the R1008 residue (R1007 in flycatcher) also resulted in a right shift of the PI(4,5)P_2_ dose response [12]. This TRP domain residue however is not in contact with PI(4,5)P_2_ in the cryoEM structure (6nr2), but rather it interacts with the menthol analogue WS12 [25]. Consistent with this, the menthol dose response in the R1008Q mutant was also right shifted [12]. Menthol was shown to increase the apparent affinity of the channel for PI(4,5)P_2_ [12] therefore the right shift in the PI(4,5)P_2_ dose response in the R1008Q mutant was likely due to an indirect effect of reduced menthol affinity.

Interestingly, the cryoEM structure of TRPM8 in the combined presence of PI(4,5)P_2_ and icilin or WS12 was still in a closed state [25], pointing to the possibility of missing additional factors, such as polyphosphate [26]. This is in sharp contrast to TRPV5 cryoEM structure, which showed an open configuration in the presence of PI(4,5)P_2_ [27]. While the proximal C-terminus, the S4-S5 linker and the pre-S1 segment contributed to PI(4,5)P_2_ binding in both structures, the location of the lipid was different in TRPM8 and TRPV5 (Figure 2).

## The role of PI(4,5)P_2_ in channel desensitization/adaptation

**Figure 3.**
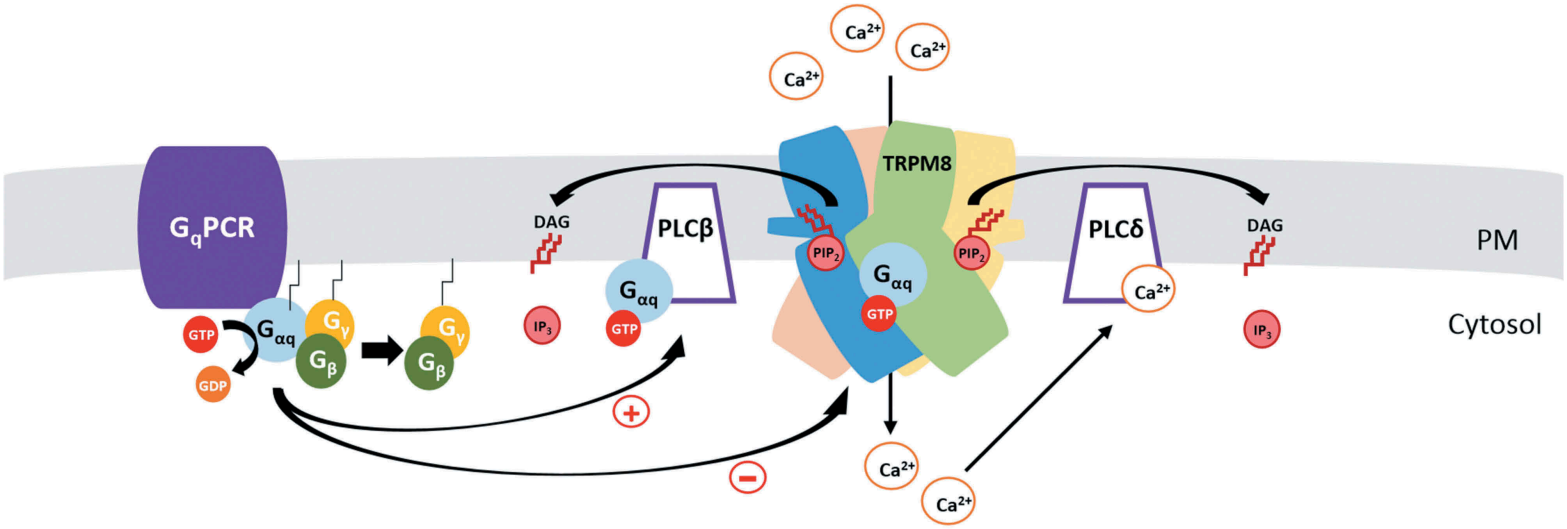
Regulation of TRPM8 by PI(4,5)P_2_ and G-proteins During GPCR activation, the G_αq_ subunit stimulates PLCβ isoforms that induce a decrease of plasma membrane PI(4,5)P_2_ levels which limits TRPM8 activity. G_αq_ also directly binds to the channel and inhibits its activity (left side of cartoon). Ca^2+^ entering through the channel stimulates PLCδ isoforms, which leads to a decrease in PI(4,5)P_2_ levels and inhibits channel activity (right side of cartoon). See main text for details.

Many Ca^2+^ permeable ion channels undergo Ca^2+^ dependent desensitization i.e. decreased activity in the continuous presence of the activator. For TRPM8 it was shown that the mechanism of desensitization is the activation of a Ca^2+^ sensitive PLC isoform by Ca^2+^ influx through the channel, and the concurrent decrease in PI(4,5)P_2_ levels (Figure 3). This model is based on the following findings. 1. As described in detail before, TRPM8 activity requires the presence of PI(4,5)P_2_. 2. Activation of TRPM8 in the presence of extracellular Ca^2+^ evokes a decrease in PI(4,5)P_2_ levels [11,12,23]. The dominant PLC isoform that is activated by Ca^2+^ influx is PLCδ4; genetic deletion of this enzyme resulted in increased cold- and menthol-induced current is DRG neurons [28]. 3. A decrease in PI(4,5)P_2_ levels by voltage activated or chemically activated lipid phosphatases (independent of PLC activation) is sufficient to inhibit TRPM8 activity [11,23,29]. 4. Interfering with PI(4,5)P_2_ depletion by supplying excess PI(4,5)P_2_ through the whole cell patch pipette decreased desensitization [23].

There are two lines of evidence suggesting a role of PLC-mediated PI(4,5)P_2_ depletion in temperature adaptation *in vivo*. First, an elegant study by the Gereau laboratory showed that withdrawal of the hind paw from cold stimuli does not depend on the absolute temperature of the stimuli, but rather on the temperature difference between the cold stimulus applied and the temperature of the glass surface the mice were standing on [30]. In other words, mice adapt to the temperature of their environment. This adaptation was significantly reduced by genetic deletion of TRPM8 and by intraplantar injection of the PLC inhibitor U73122 [30]. These data indicate that PLC mediated desensitization of TRPM8 plays a role *in* *vivo* in temperature adaptation. Second, as describe before, the main PLC isoform activated by Ca^2+^ influx in DRG neurons was identified to be PLCδ4. PLCδ4^−/-^ mice showed increased sensitivity of evaporative cold, but not to noxious heat and to mechanical stimuli [28].

## TRPM8 inhibition by G_q_-coupled receptors; the roles of PI(4,5)P_2_, G_αq_, and PKC

Activation of G_q_-coupled receptors, that activate PLCβ isoforms, such as the bradykinin B2 receptor, was also shown to inhibit TRPM8 activity. In contrast to the general agreement on the involvement of PI(4,5)P_2_ depletion in Ca^2+^ dependent desensitization, the involvement of this lipid in receptor-induced TRPM8 inhibition has been debated.

Both publications that originally described PI(4,5)P_2_ dependence of TRPM8 activity found that activation of cell surface receptors that couple to PLC inhibited TRPM8 activity in expression systems, and both papers attributed it to PI(4,5)P_2_ depletion [12,21]. Recombinant TRPM8 channels expressed in Xenopus oocytes were inhibited by activation or the receptor tyrosine kinase PDGF receptor that couples to PLCγ [12]. Mutations in the PDGFR that prevent PLC activation eliminated TRPM8 inhibition. Mutation of putative PI(4,5)P_2_ interacting residues in TRPM8 that decreased apparent affinity for PI(4,5)P_2_ increased the level of inhibition [12]. Consistent with these results, Liu et al found that activation of both NGF receptors that activate PLCγ and M1 muscarinic receptors that activate PLCβ inhibited menthol-induced TRPM8 currents [21].

A publication shortly after these two papers showed that menthol-induced Ca^2+^ responses in DRG neurons were inhibited by bradykinin, and the effect was eliminated by the PKC inhibitor Bisindolylmaleimide (BIM) [31]. High concentrations of PKC activating phorbol esters, PDBu and PMA (1 μM) inhibited TRPM8 activity, and both the effects of phorbol esters and bradykinin were inhibited by the protein phosphatase inhibitor okadaic acid, therefore it was concluded that TRPM8 is inhibited by PKC-mediated dephosphorylation upon bradykinin receptor activation [31].

A later publication postulated a novel alternative mechanism of inhibition by direct binding of G_αq_ to TRPM8, and challenged the roles of both PKC and PI(4,5)P_2_ depletion [16]. The key findings of this manuscript supporting inhibition by direct binding of G_αq_ are the following. The authors replaced a small segment of G_αq_ with that of G_αi_ and found that this chimera (G_αqiq_) was deficient in activating PLC. Coexpression of a constitutively active mutant (Q209 L) of this G_αqiq_ chimera was sufficient by itself to inhibit TRPM8 activity. The authors also found that G_αq_ binds to TRPM8, and application of purified G_αq_ to excised inside out patches inhibited TRPM8 activity in the presence of PI(4,5)P_2_ [16]. A follow up publication found that G_α11_ was much less efficient than G_αq_ in inhibiting TRPM8 [32], despite that G_α11_ is similarly efficient to G_αq_ in stimulating PLC.

Zhang et al also found that the PKC inhibitor BIM did not reduce the inhibitory effect of bradykinin, and PMA did not inhibit TRPM8 activity, arguing against the role of PKC [16]. The authors listed two arguments against the involvement of PI(4,5)P_2_. 1. The PLC inhibitor U73122 had no effect on bradykinin-induced inhibition of TRPM8, but it partially reduced the inhibitory effect of activating G_q_-coupled (H1R) histamine receptors. 2. Mutations in putative PI(4,5)P_2_ binding residues in the TRP domain did not eliminate histamine-induced inhibition of TRPM8 [16]. Mutation of these residues however is expected to increase inhibition, by reducing apparent affinity for PI(4,5)P_2_ [12], and indeed the histamine-induced inhibition of the K995Q mutant was somewhat larger than that observed in wild-type channels [16].

A subsequent paper by Liu et al provided evidence that decreased levels of PI(4,5)P_2_ are involved in receptor-induced inhibition of TRPM8 activity [17]. This work demonstrated that PI(4,5)P_2_ levels decrease in DRG neurons upon activation of endogenous G_q_-coupled receptors using two different fluorescence-based PI(4,5)P_2_ sensors. The PI(4,5)P_2_ decrease in TRPM8 positive neurons was more pronounced than that observed in TRPM8 negative neurons. To stimulate endogenous G_q_-coupled receptors a mixture of pro-inflammatory agonists was used, as no single receptor was expressed in all TRPM8 positive neurons. Consistent with earlier data, PI(4,5)P_2_ levels also decreased in a recombinant system upon activation of G_q_-coupled receptors. This decrease was larger in the presence of extracellular Ca^2+^ than in its absence, which correlated well with the larger inhibition in the presence of extracellular Ca^2+^ compared to nominally Ca^2+^ free conditions. Inclusion of PI(4,5)P_2_ in the whole cell patch pipette decreased inhibition by G_q_-coupled receptor activation both in DRG neurons and in an expression system [17]. Cumulatively, these data provided strong support for the involvement of PI(4,5)P_2_ depletion in TRPM8 inhibition upon activation of G_q_-coupled receptors.

Liu et al [17] also confirmed that coexpressing the constitutively active Q209 L-G_αqiq_ construct used by Zhang et al [16] inhibited menthol-induced TRPM8 activity. Co-transfecting Q209 L-G_αqiq_ with TRPM8 also rendered the channel more sensitive to inhibition by decreasing PI(4,5)P_2_ levels by the voltage activated phosphoinositide phosphatase ciVSP [17]. Overall Liu et al concluded that the decrease of PI(4,5)P_2_ and direct binding of G_αq_ synergize in inhibiting TRPM8 activity (Figure 3).

**Figure 4.**
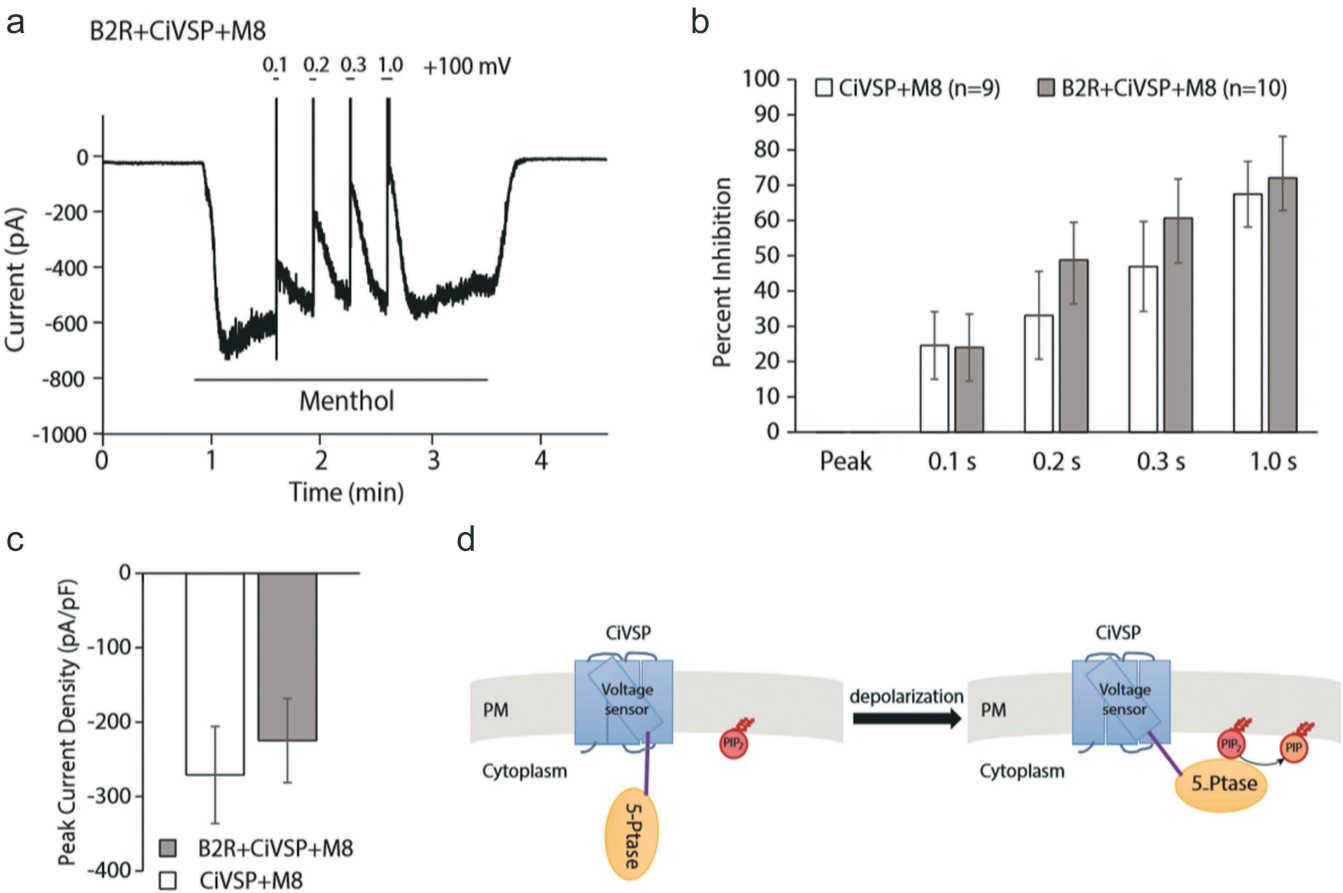
Bradykinin receptor co-expression does not interfere with TRPM8 inhibition by the voltage sensitive phosphoinositide phosphatase ciVSP. (a). Representative trace showing menthol-induced TRPM8 activity in HEK293 cells transfected with TRPM8, ciVSP, and B2 bradykinin receptors. The holding potential was −60 mV, channel activity was evoked by the application of 500 μM menthol in the absence of extracellular Ca^2+^ to avoid desensitization, ciVSP was activated by depolarizing pulses to 100 mV for 0.1, 0.2, 0.3 and 1 s. The experiment was performed the same way as shown in Figure 10 of Liu et al [17]. (b). Summary of the data, channel inhibition by depolarizing voltage pulses is shown for cells transfected with B2 receptors, TRPM8 and ciVSP, and for cells transfected with TRPM8 and ciVSP. (c). Summary of current densities (d). Cartoon explaining ciVSP.

Another paper by Zhang [18], published at the same time as Liu et al [17] made a case that PI(4,5)P_2_ depletion is not involved in bradykinin receptor mediated inhibition of TRPM8, and proposed that direct inhibition by G_αq_ is the sole mechanism. The key findings of the article [18] are the following: Genetic deletion of G_αq_ eliminated inhibition of TRPM8 in DRG neurons, but potentiation of TRPV1 currents remained intact after bradykinin receptor activation. Mutating G_αq_ interacting residues in TRPM8 eliminated inhibition by bradykinin receptor activation, but the channel was still inhibited by depleting PI(4,5)P_2_ with a chemically inducible phosphatase. Additionally, co-expression of the bradykinin B2 receptor rendered the channel resistant to inhibition by chemically induced depletion of PI(4,5)P_2_ [18]. In our hands, co-expression of B2 bradykinin receptors did not interfere with inhibition of TRPM8 activity by decreasing PI(4,5)P_2_ using the voltage dependent lipid phosphatase ciVSP (Figure 4). This shows that the ability of the B2 receptor to interfere with inhibition of TRPM8 by decreasing PI(4,5)P_2_ may be specific to the chemically inducible phosphatase system.

The proposed exclusive role of inhibition by G_αq_ was specific to B2 bradykinin receptors, as mutating G_αq_ binding residues did not eliminate TRPM8 inhibition by H1 histamine receptors, and TRPM8 was inhibited by chemically induced PI(4,5)P_2_ depletion in the presence of H1 histamine receptors [18].

## Conclusions

The plasma membrane phospholipid PI(4,5)P_2_ is an important cofactor for TRPM8. The recent cryoEM structure of TRPM8 with PI(4,5)P_2_ provides molecular level understanding of how this lipid binds to TRPM8. Ca^2+^ influx through TRPM8 activates a Ca^2+^ sensitive PLCδ isoforms leading to decreased PI(4,5)P_2_ levels and concurrently decreased channel activity. This Ca^2+^ dependent desensitization leads to adaptation to lower temperatures *in vivo*. Upon activation of G_q_-coupled receptors, both decreased PI(4,5)P_2_ levels and direct binding of G_αq_ plays a role in channel inhibition in a synergistic fashion, but the relative contributions of the individual pathways is likely receptor specific.
